# Targeting ARF6-SUCNR1 Axis: Antisense Oligonucleotide Adjuvants for Neutrophil Immunometabolism

**DOI:** 10.3390/ijms27157007

**Published:** 2026-08-04

**Authors:** Yangyang Wang, Ye Chen

**Affiliations:** Jilin Collaborative Innovation Center for Antibody Engineering, Jilin Medical University, Jilin 132013, China

**Keywords:** ARF6, SUCNR1, ASO, vaccine adjuvant, neutrophil, immunometabolism, inflammation, immune response

## Abstract

Conventional vaccine adjuvants often lack cell-type specificity and readily trigger excessive systemic inflammation, limiting their clinical application. Immunometabolic signaling centered on the ARF6 (ADP-ribosylation factor 6)-SUCNR1 (Succinate receptor 1) axis governs neutrophil recruitment and subsequent B-cell activation at vaccination sites, offering a promising target to balance adjuvant potency and biosafety. Separately published single-gene data confirm two opposing functions for the two mediators: ARF6 overactivation amplifies local pathological inflammation, whereas succinate-stimulated SUCNR1 drives neutrophil infiltration and humoral immune priming. However, direct paired evidence verifying their bidirectional crosstalk within immunization microenvironments remains limited. This review systematically integrates current research on ARF6 and SUCNR1 immunometabolism to outline a hypothetical dual-regulatory adjuvant strategy: local, transient partial silencing of pathological ARF6 activity via ARF6-targeted ASOs (antisense oligonucleotides) paired with localized succinate delivery to sustain protective SUCNR1 signaling. We summarize the molecular basis of this “one inhibition, one activation” paradigm, alongside ASO chemical modification, myeloid-targeted design, and nanocarrier delivery solutions that resolve the unique ARF6 endocytosis paradox. We further dissect unresolved translational risks, including concentration-dependent succinate inflammatory effects, carrier immunogenicity, and potential impairment of baseline neutrophil migration upon ARF6 suppression. Overall, this work synthesizes fragmented mechanistic data to construct a testable neutrophil-centered adjuvant framework, and highlights outstanding technical and biosafety hurdles that require dedicated preclinical validation to support future adjuvant development.

## 1. Introduction

Immunometabolism has gradually become a core research direction in vaccinology, inflammation and immunotherapy over the past five years [1,2,3]. Traditional vaccine adjuvants often lack cell specificity and easily induce excessive systemic inflammation, which restricts their clinical application [4]. Therefore, exploring precise regulatory strategies based on metabolic–immune axes has become an urgent demand for novel adjuvant development.

ARF6, a small GTPase regulating membrane trafficking and cytoskeletal dynamics, is widely expressed in metabolic and immune cells [5,6,7]. Excessive ARF activation amplifies local inflammation and impairs normal immune cell function, acting as a negative regulator in vaccine microenvironments [5,6,8]. SUCNR1, a G-protein-coupled receptor sensing extracellular succinate, can recruit neutrophils and activate B cells via the SUCNR1-IRF5-BAFF pathway, exerting adjuvant activity [9]. Accumulating evidence proves that ARF6 and SUCNR1 interact closely to jointly govern neutrophil behavior [7,10], forming a key immunometabolism axis linking metabolism and adaptive immunity.

Current intervention approaches mainly rely on small-molecule inhibitors for the universal suppression of signaling axes, which cause severe off-target effects due to the essential physiological functions of ARF6 and SUCNR1 in normal tissues [9,11]. To address this dilemma, we propose a differentiated regulation strategy for the ARF6-SUCNR1 axis in vaccine design: applying ARF6-targeted antisense oligonucleotides (ASOs) to specifically inhibit pathological ARF6 overactivation, while using succinate to maintain and activate SUCNR1-mediated immune cascades. This “one inhibition, one activation” mode can effectively suppress adverse inflammation while maximizing the adjuvant effect of SUCNR1, which is completely different from conventional single-target inhibition schemes.

**Figure 1:**
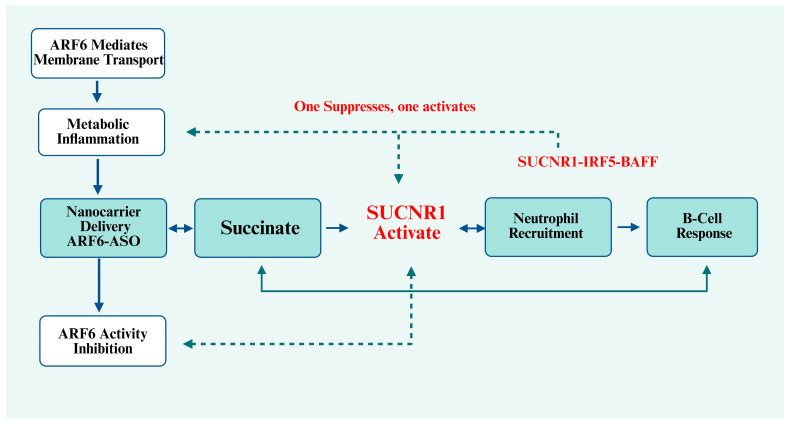
Diagram of ARF6-SUCNR1 Immunometabolism Synergy Adjuvant Regulating Immune Balance. The ARF6-SUCNR1 immunometabolism axis, using a nanocarrier to deliver ARF6-ASO combined with succinate, tackles the ARF6 endocytosis paradox through a ‘suppressing ARF6 over-inflammation while activating the SUCNR1-IRF5-BAFF immune pathway’ approach, balancing vaccine immunogenicity and biosafety. Solid arrows = validated evidence; dashed arrows = unconfirmed hypothetical interactions.

Current investigations of ARF6 and SUCNR1 are largely isolated, and their immunometabolic crosstalk alongside the pros and cons of the dual-modulation adjuvant design remains unsynthesized. By compiling recent evidence of ARF6-driven inflammatory metabolism and SUCNR1-mediated neutrophil immunity, this review constructs a unified mechanistic framework of the ARF6–SUCNR1 axis, objectively evaluating the feasibility and hidden risks of the proposed regulatory strategy rather than unilaterally advocating its clinical translation. We dissect reciprocal signaling between the two molecules, summarize the design and nanocarrier delivery of ARF6-specific ASOs, and analyze both the strengths and translational barriers of the “ARF6 inhibition + SUCNR1 activation” model, offering a balanced mechanistic reference for developing low-toxicity neutrophil-targeted vaccine adjuvants (Figure 1).

## 2. Immunometabolism: The Intersection of Metabolism, Inflammation and Vaccine Adjuvant Development

**Figure 2:**
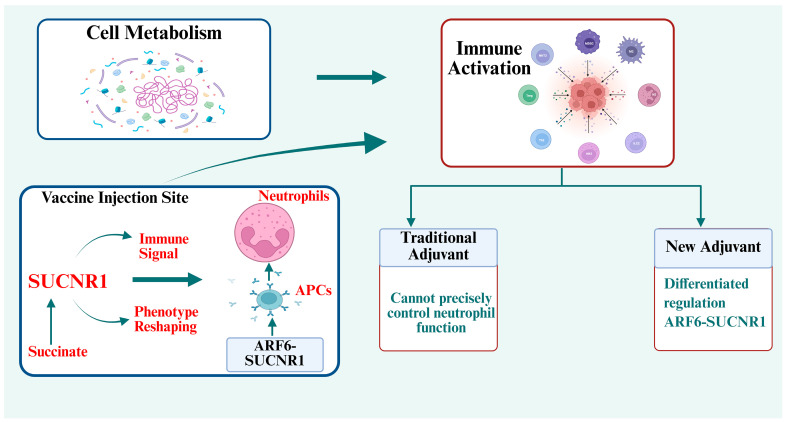
Metabolism—A next-generation adjuvant under immunology that regulates neutrophils via ARF6-SUCNR1. Immunometabolism links cell metabolism with immune activation. Succinate regulates neutrophils in core effector cells through the ARF6-SUCNR1 axis.

Immunometabolism bridges cellular metabolism and immune activation. In vaccination sites, accumulated metabolites such as succinate act as immune signals to reshape immune cell phenotypes [12,13]. Neutrophils, as atypical antigen-presenting cells, dominate the adjuvant-induced humoral immunity and become the core effector cells of the ARF6-SUCNR1 axis [14,15]. Traditional adjuvants cannot precisely regulate neutrophil function and balance immune response and inflammation [16,17,18,19]. Targeting the ARF6-SUCNR1 axis with differentiated regulation provides a new solution for next-generation adjuvants (Figure 2).

### 2.1. Biological Functions of ARF6 and SUCNR1: Two Opposite Regulators in Vaccine Microenvironment

**Figure 3:**
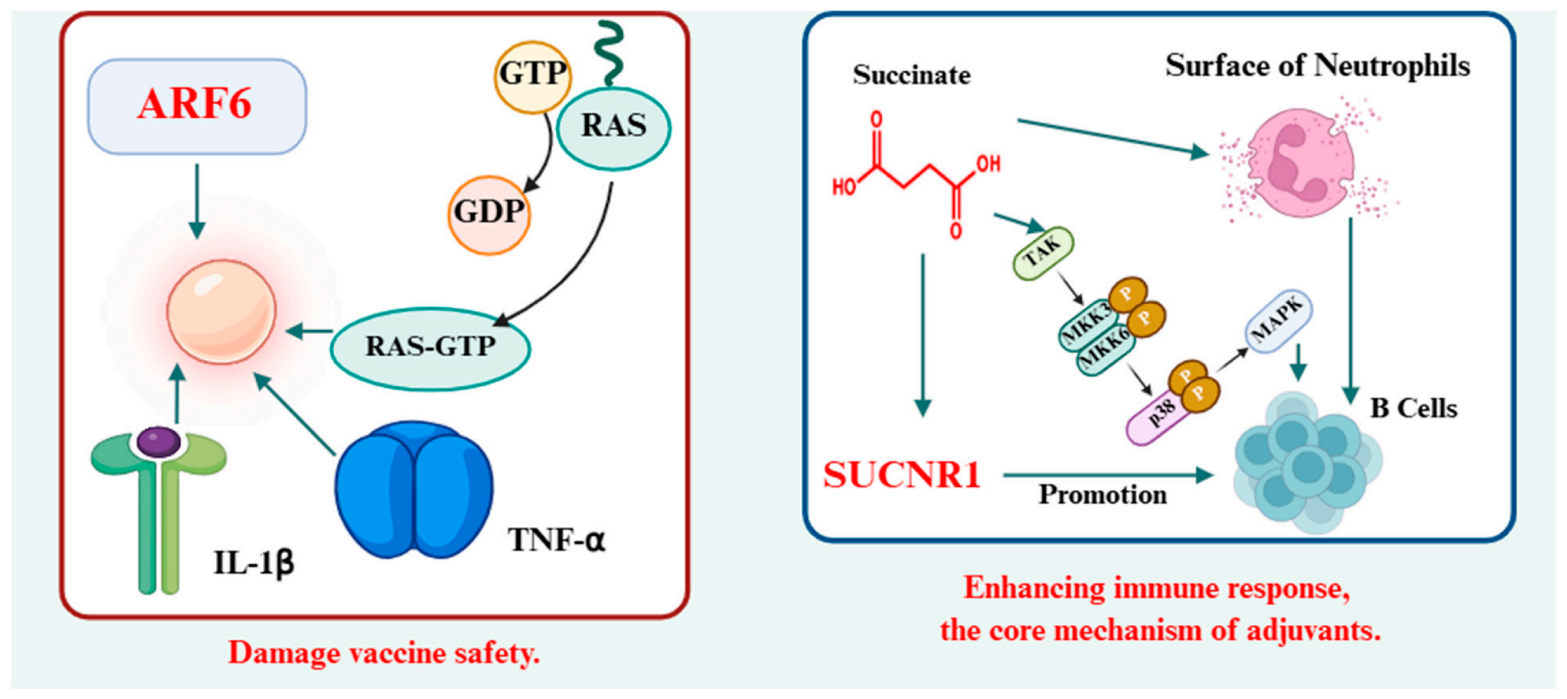
The two-way antagonistic regulation function of ARF6 and SUCNR1 in the vaccine microenvironment. In the situation, ARF6 mediates harmful inflammation while SUCNR1 mediates strong immune responses, and their opposite effects support the idea of a ‘one suppresses, one boosts’ approach to adjuvant regulation.

ARF6 is a negative factor in vaccine microenvironments [20]. Its overactivation disrupts membrane homeostasis and amplifies unnecessary inflammatory responses, which damages vaccine safety [21,22,23]. In contrast, SUCNR1 acts as a positive immune regulator: binding with succinate triggers neutrophil recruitment and B-cell activation, which is the core mechanism of succinate as a potent adjuvant [9,10,24] (Figure 3). The opposing roles of ARF6 and SUCNR1 lay the foundation for the “one inhibition, one activation” regulatory strategy.

#### 2.1.1. ARF6 Signaling Axis: Membrane Trafficking and Pro-Inflammatory Effects

As a small GTPase, ARF6 cycles between GDP-bound inactive state and GTP-bound active state, regulating vesicle transport and actin remodeling [25,26]. In pancreatic β-cells and peripheral tissues, ARF6 maintains glucose homeostasis [27,28]; while in immune cells within vaccination sites, excessive ARF6 activation promotes inflammatory receptor recycling and amplifies local low-grade inflammation [21,29].

**Figure 4:**
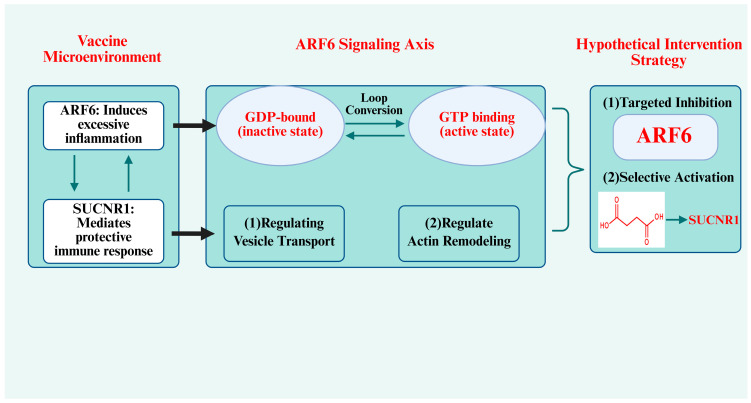
Function of the ARF6 and SUCNR1 Signaling Axis and Targeted Intervention Strategies. ARF6 triggers excessive inflammation while SUCNR1 mediates protective immunity; ARF6 regulates vesicle transport and actin remodeling through the GDP/GTP cycle.

Of note, ARF6 executes indispensable homeostatic physiological functions under baseline conditions: it maintains glucose metabolism homeostasis in pancreatic β-cells and mediates constitutive physiological membrane recycling across all immune cell subsets, which are essential for routine immune surveillance [20,27]. The pathological detrimental phenotype only emerges when ARF6 undergoes sustained excessive GTP loading at vaccination sites post-adjuvant administration [19]. Our proposed ASO design hypothetically aims to induce partial, tissue-restricted, transient ARF6 suppression rather than permanent full protein ablation. However, uncontrolled ARF6 activity will cause adverse reactions such as local redness and swelling after vaccination [20,30], which impairs vaccine safety. Therefore, moderate and specific inhibition of ARF6 is required for vaccine optimization (Figure 4).

Notably, basal low-level ARF6 activity is indispensable for routine immune cell homeostasis, sustaining steady SUCNR1 membrane recycling and baseline neutrophil migration capacity [31,32]. Only sustained, abnormally elevated ARF6 GTP loading triggered by succinate downstream signaling drives destructive inflammatory amplification [31]. Our ARF6-targeted ASO does not completely erase ARF6 expression, but achieves partial transient knockdown limited to myeloid cells at injection sites, which separates pathological ARF6 overactivation from its necessary physiological functions [28].

#### 2.1.2. SUCNR1 Signaling Axis: Succinate Sensing and Adjuvant Activity

Different from the pro-inflammatory role of ARF6, the SUCNR1 signaling axis is the core pathway to exert adjuvant effects. Succinate, as a natural metabolite, activates SUCNR1 to initiate neutrophil recruitment and B-cell activation, which is the basis for enhancing vaccine immunity [9,33]. In vaccine design, we need to retain and fully activate SUCNR1 signaling instead of inhibiting it. Traditionally, extracellular succinate specifically binds to membrane SUCNR1 and activates the downstream Gq signaling pathway, regulating the cell polarization, migration and cytokine secretion of immune cells [31,34]. In metabolic inflammatory tissues, the accumulation of succinate activates SUCNR1 in macrophages, promotes macrophage polarization to a pro-inflammatory phenotype, and increases the secretion of chemokines and pro-inflammatory factors [34,35,36]. As a bridge connecting metabolism and immunity, the succinate–SUCNR1 axis can convert metabolic stress signals into immune activation signals, and participate in the occurrence of various inflammatory diseases (Figure 4).

Despite its adjuvant potency, persistent, systemic SUCNR1 hyperactivation drives multiple inflammatory disorders, including neutrophil-mediated acute respiratory distress syndrome (ARDS) and autoimmune uveitis via enhanced neutrophil extracellular trap (NET) release [10,24]. In our local vaccination setting, succinate is co-delivered with ARF6-ASO exclusively at the injection microenvironment, rather than administered systemically. Transient partial ARF6 silencing interrupts the SUCNR1-ARF6 positive feedback loop that amplifies unrestrained inflammation, thereby separating the beneficial B-cell priming cascade from excessive tissue-damaging neutrophil activation triggered by sustained SUCNR1 signaling [9,31].

Notably, succinate exerts strictly concentration- and time-dependent divergent effects on myeloid cells. Low, localized succinate doses at injection sites selectively drive CXCL2 secretion and limited neutrophil recruitment for humoral priming [9,13]; prolonged high succinate exposure or systemic metabolite leakage shifts macrophage polarization toward pro-inflammatory M1 subsets and promotes excessive NET release [37,38]. Individuals with underlying metabolic disorder or chronic inflammatory comorbidities carry chronically elevated baseline circulating succinate [39,40]; local vaccine-delivered succinate may compound systemic metabolic inflammation if leakage from the injection niche occurs [9,40]. Even with intramuscular local delivery, minor vascular absorption of succinate cannot be fully excluded, raising off-target metabolic perturbation risks in vulnerable patient populations [9,39].

**Figure 5:**
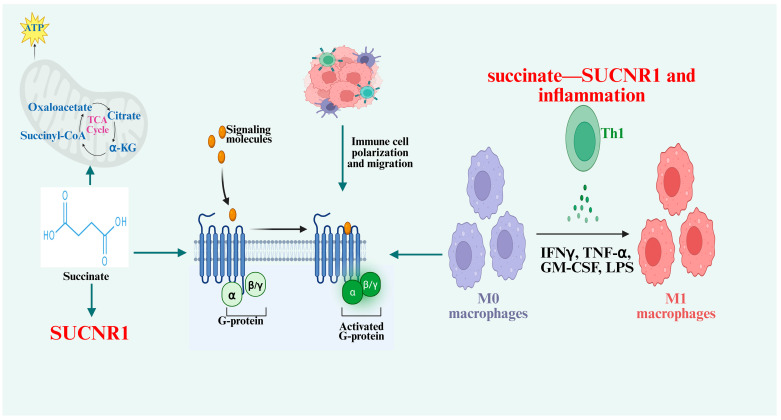
Succinate–SUCNR1 regulates the pro-inflammatory polarization pathway of macrophages. Succinate binds to SUCNR1 to activate the Gq pathway, driving macrophages toward a pro-inflammatory state. This axis acts as a metabolic-immune crosslink point, converting metabolic stress signals into immune activation signals, and is involved in inflammatory lesions.

SUCNR1 mediates neutrophil-associated immune activation signals. In vaccine immunization sites, succinate formulated in vaccines activates SUCNR1 in resident macrophages and significantly upregulates the expression and secretion of chemokine CXCL2 [9,41]. CXCL2 is a classic neutrophil chemokine, which can rapidly recruit a large number of circulating neutrophils to local immunization sites [42,43]. The neutrophils recruited to immunization sites are the main source of B-cell-activating factor (BAFF) to activate CD19^+^ B cells in local tissues and spleen, upregulate the expression of B-cell activation markers CD69 and CD40, and promote the differentiation of B cells into antibody-secreting cells [9,44]. Further mechanism studies show the SUCNR1-IRF5-BAFF signaling cascade (Figure 5). This pathway is the molecular basis for succinate to exert adjuvant activity.

### 2.2. Crosstalk Between ARF6 and SUCNR1 Axes

**Figure 6:**
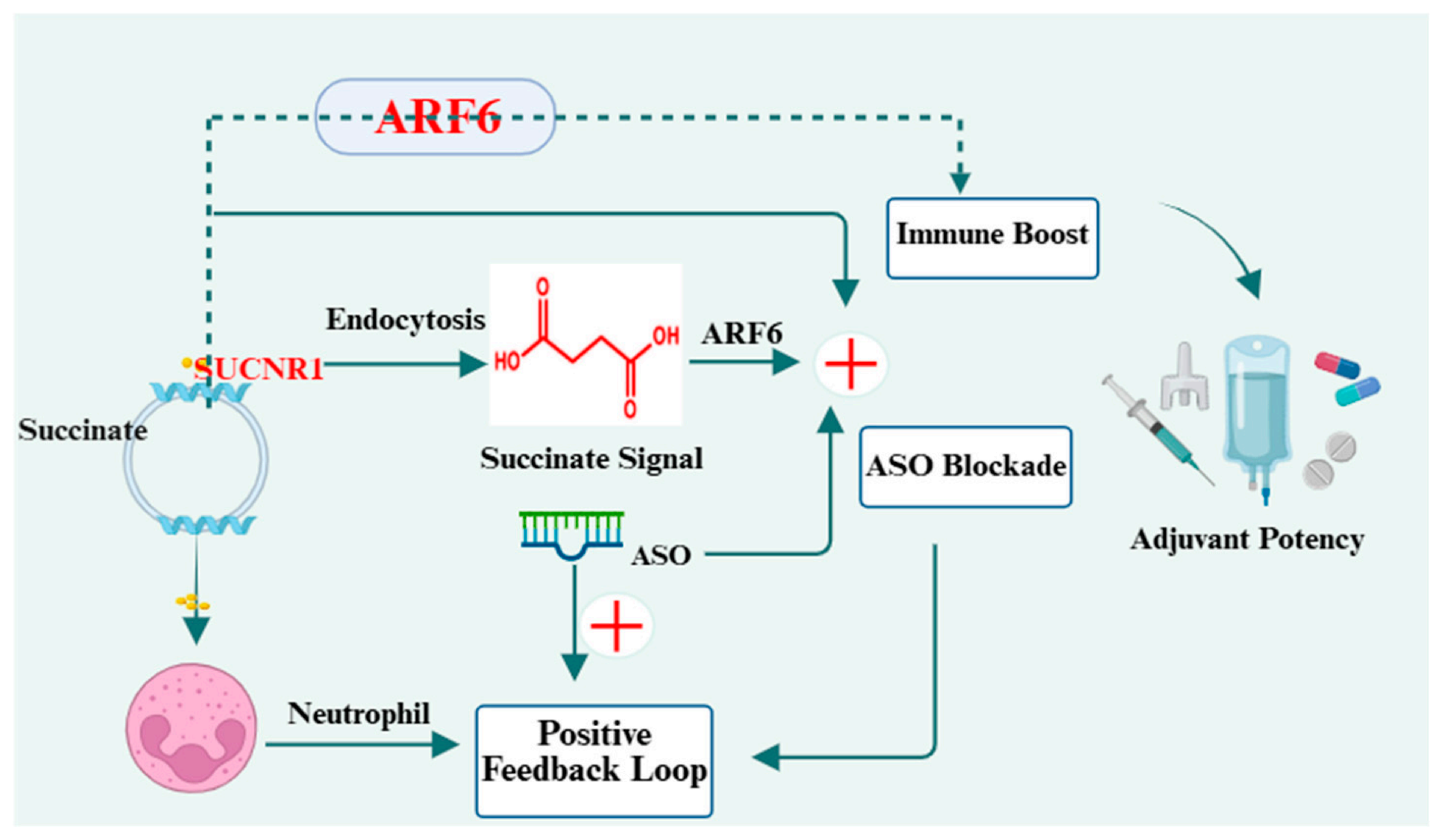
Cross-talk of ARF6 and SUCNR1 axis in immune cells and adjuvant optimization plan. The interaction mechanism where ARF6 and SUCNR1 form a pro-inflammatory positive feedback loop, and the adjuvant optimization strategy using ASOs to specifically block ARF6 while keeping SUCNR1 immune enhancement. Solid arrows = validated evidence; dashed arrows = unconfirmed hypothetical interactions.

ARF6 and SUCNR1 are closely coupled in immune cells. ARF6 sustains SUCNR1 membrane recycling (indirect single-gene evidence). The downstream positive feedback loop boosting ARF6 activity remains an unvalidated hypothesis without dual-gene verification. This proposed crosstalk explains concurrent ARF6 overactivation upon SUCNR1 stimulation at injection sites [31]. Accordingly, we put forward a testable hypothetical intervention: partial silencing of the pro-inflammatory ARF signaling via ASOs while preserving succinate-triggered SUCNR1 immune cascades, which may interrupt the detrimental feedback loop without dampening adjuvant potency; this model awaits paired dual-target in vivo verification (Figure 6).

### 2.3. Co-Regulation of Neutrophil Biology by ARF6-SUCNR1 Axis

**Figure 7:**
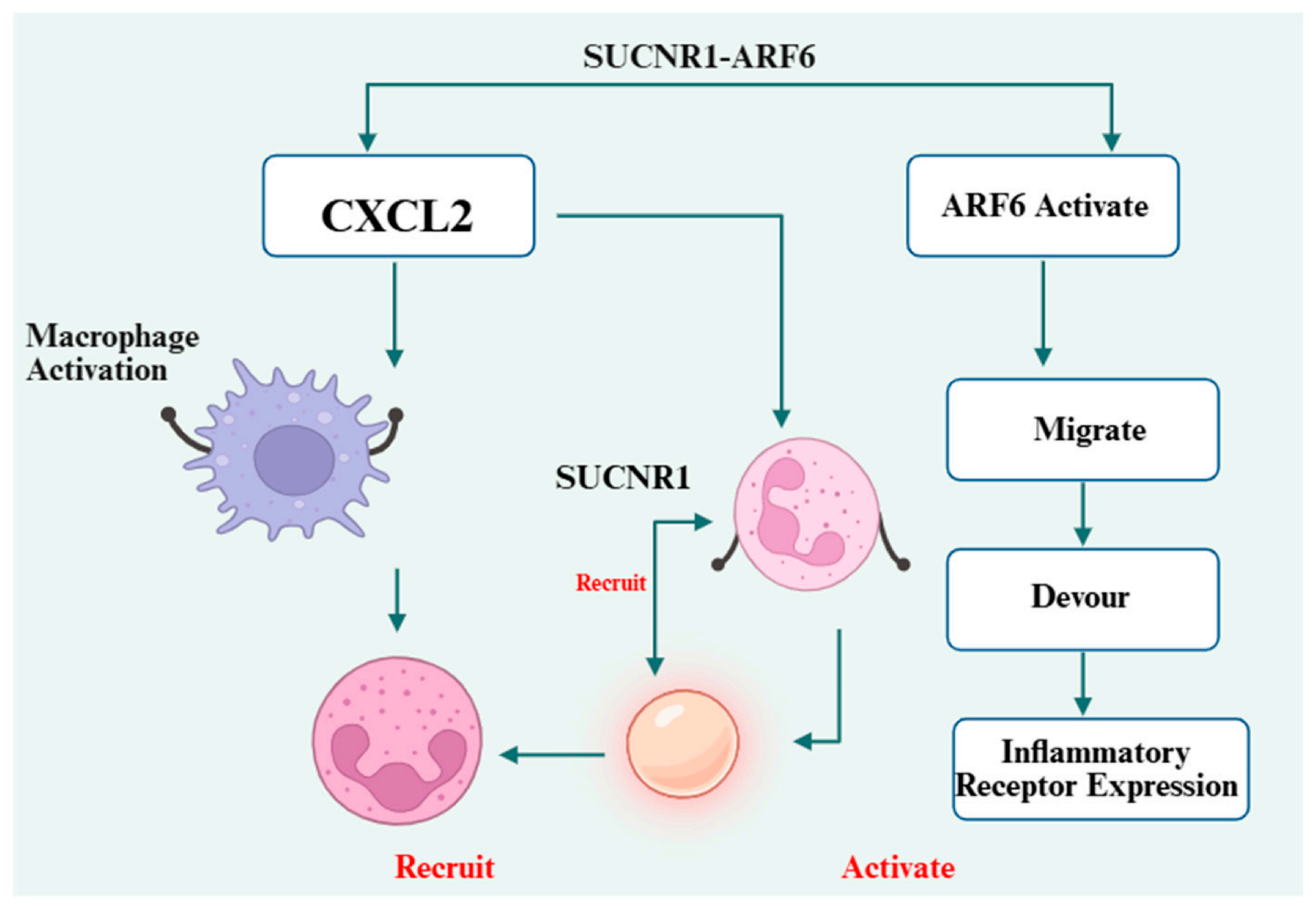
Diagram of ARF6-SUCNR1 Axis Coordinating Neutrophil Recruitment and Activation. Activated SUCNR1 signaling recruits neutrophils to inflammation sites through macrophage CXCL2, while ARF6 activates neutrophils by regulating their migration, phagocytosis, and inflammatory receptor expression. Together, they work in coordination to control neutrophil immune responses.

Neutrophils are the key effector cells of the ARF6-SUCNR1 axis [7,10,24]. SUCNR1 dominates the directional recruitment of neutrophils to inflammatory sites through macrophage CXCL2; while ARF6 controls the migration, phagocytosis and receptor function of neutrophils after arrival at the lesion [7,42,45,46]. During neutrophil chemotaxis, ARF6 regulates actin remodeling at the leading edge of cells, providing power for cell migration [7]. Inhibition of ARF6 will significantly reduce the chemotactic ability of neutrophils, even if SUCNR1 signal is activated and CXCL2 is highly expressed (Figure 7).

In addition, ARF6 maintains the surface expression of inflammatory receptors and BAFF-related receptors on neutrophils through membrane recycling, ensuring that neutrophils can continuously receive external inflammatory signals and secrete BAFF [7,44,45]. The two axes divide labor and cooperate: SUCNR1 is responsible for “recruiting” neutrophils, and ARF6 is responsible for “activating” neutrophils, which jointly maximize the immune effect of neutrophils.

### 2.4. Shared Downstream Effectors: Membrane Trafficking and Cytoskeletal Remodeling

Both axes converge on membrane trafficking and cortical actin remodeling, based entirely on separate single-gene indirect experimental data [31]. Individually published work confirms ARF6 supports general GPCR membrane recycling [20], and independent SUCNR1 studies show receptor internalization upon ligand binding [31,36]; however, direct co-detection data proving ARF6 specifically recycles internalized SUCNR1 to prolong succinate signal duration and block lysosomal degradation remains absent.

**Figure 8:**
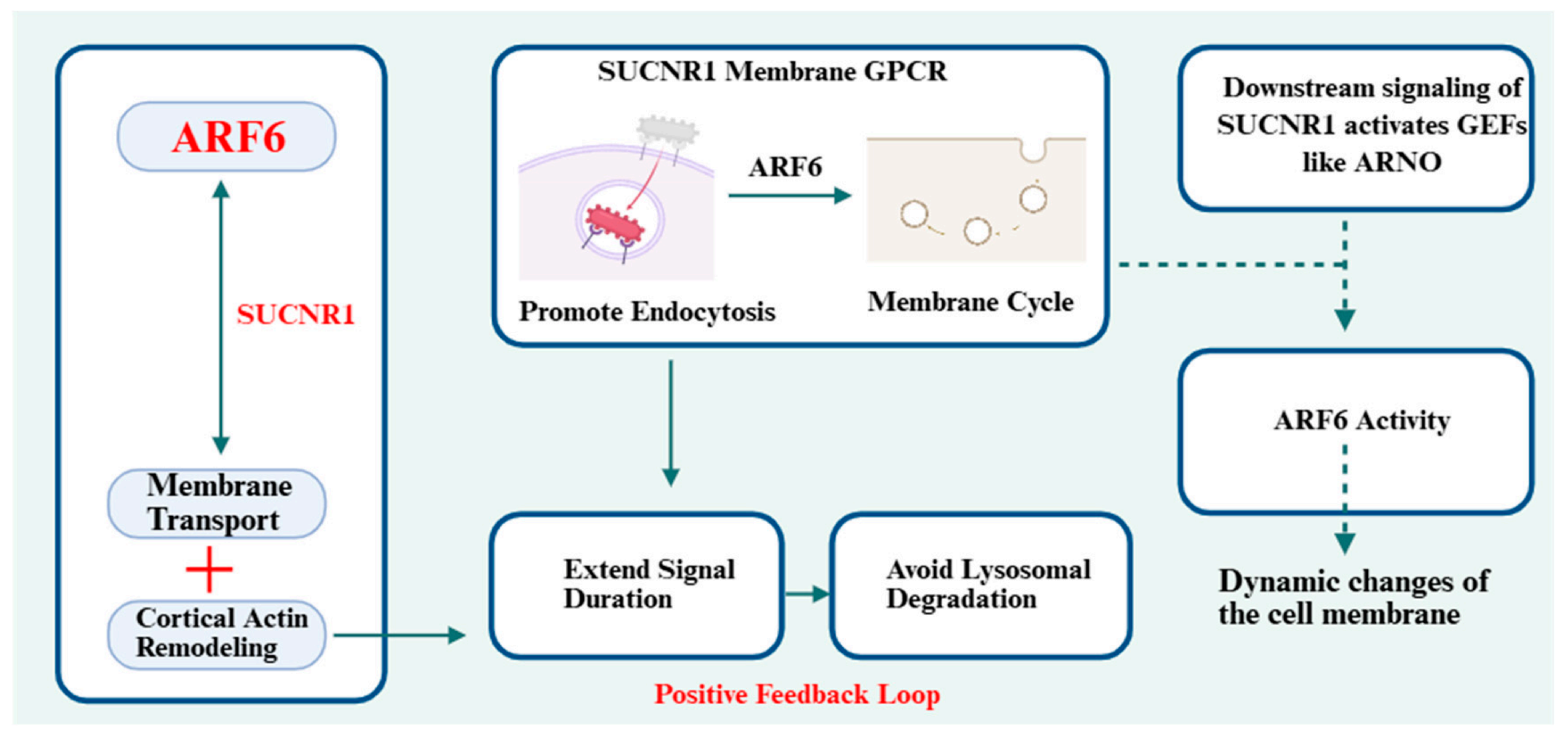
ARF6-SUCNR1 Positive Feedback Signal Regulating Cell Membrane Homeostasis Diagram. ARF6 and SUCNR1 form a positive feedback loop that jointly regulates membrane trafficking, cortical actin remodeling, and cell membrane dynamics. SUCNR1 avoids lysosomal degradation and prolongs signaling through ARF6-mediated endocytic recycling; activated SUCNR1 also triggers ARNO-type GEFs downstream, boosting ARF6 activity and amplifying the signal in both directions. Solid arrows = validated evidence; dashed arrows = unconfirmed hypothetical interactions.

The proposed positive feedback cascade where SUCNR1-downstream ARNO GEFs boost ARF6 activity is a purely hypothetical crosstalk pathway with no simultaneous ARF6/SUCNR1 co-stimulation experiments to validate bidirectional signal amplification (Figure 8). This physical connection makes separate regulation of the two axes necessary for balanced immunity.

## 3. ASOs: A Precision Tool for Targeting ARF6-SUCNR1 Axis

Small-molecule pan-inhibitors cannot distinguish ARF6 and SUCNR1, and the simultaneous suppression of both will completely abolish vaccine immunity. Antisense oligonucleotides, as precise gene silencing tools, are ideal for selective inhibition of ARF6. In this adjuvant system, we only use ARF6-targeted ASOs, and never apply SUCNR1-ASO, so as to ensure SUCNR1 can be fully activated by succinate. This targeted design may match the “inhibit ARF6 + activate SUCNR1” strategy.

### 3.1. Principles and Advantages of Antisense Oligonucleotide Therapeutics

ASO achieves single-gene specific silencing, which enables us to only suppress ARF6 without affecting SUCNR1 expression. ASO is a single-stranded nucleic acid fragment designed according to the target gene sequence [47,48]. It binds to target mRNA through base complementary pairing, induces mRNA degradation, or inhibits ribosome translation, thereby blocking the expression of target protein [49,50]. Compared with small-molecule drugs, ASO has three core advantages: first, high sequence specificity, which can accurately act on target genes and reduce off-target toxicity [51]; second, flexible design, which can be modified chemically to improve stability and tissue affinity; third, wide applicability, which can target “undruggable” targets that are difficult to bind by small molecules [52,53]. In recent years, ASO has been successfully applied in the treatment of inflammation, tumors and metabolic diseases, and its safety and effectiveness have been effectively verified [54]. Early research has proven that sequence-specific oligonucleotides can remodel local immune microenvironments to boost vaccine potency, while also highlighting common translational hurdles including poor tissue bioavailability and non-specific silencing [55]. Distinct from previous ASOs that only suppress immune suppressors, our work combines gene silencing with metabolite receptor activation to bidirectionally regulate immunometabolism, representing an unexplored design logic for adjuvant development [28].

### 3.2. Rationale for Developing ARF6-Targeted ASO as Vaccine Adjuvants

**Figure 9:**
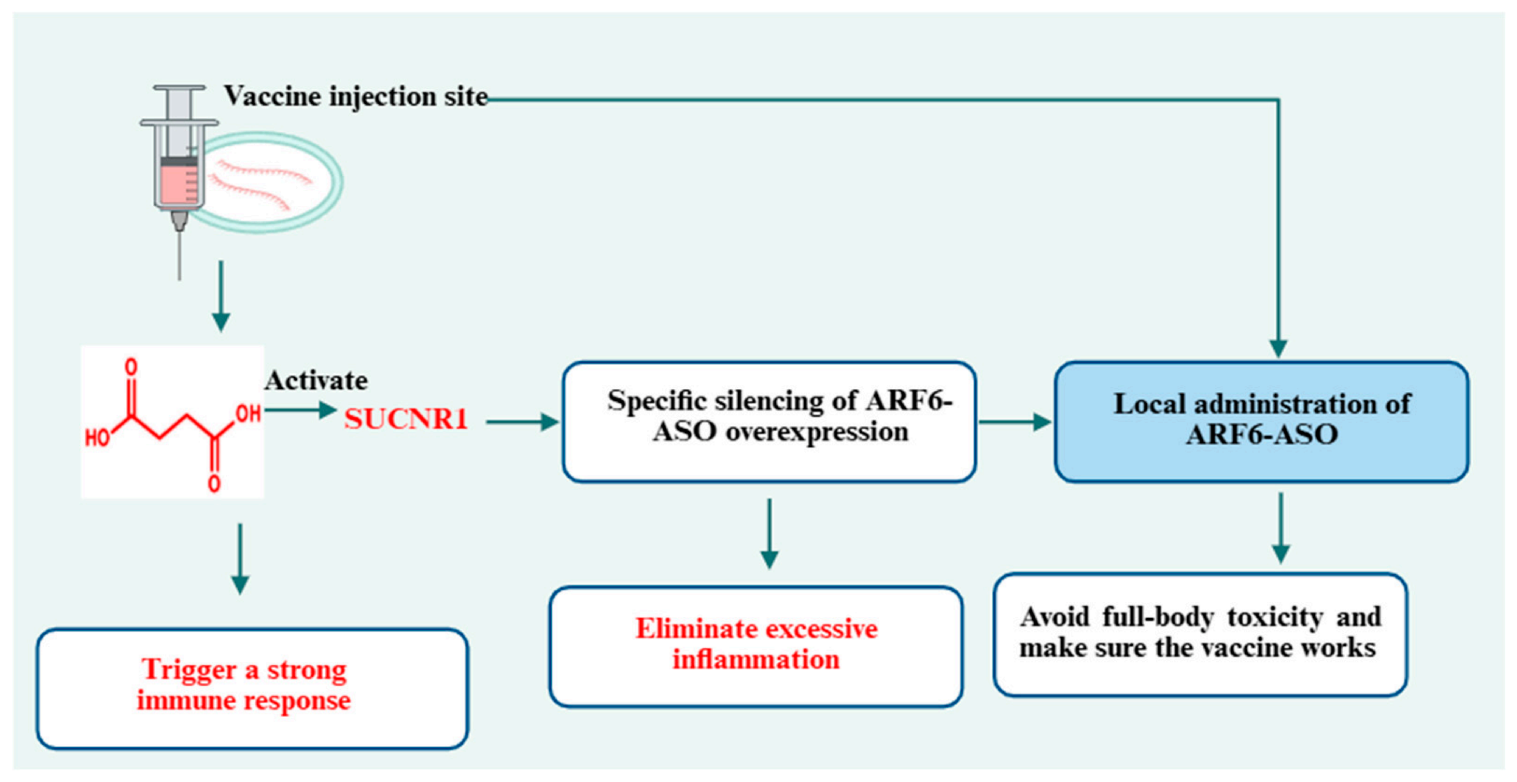
Design of ASO Vaccine Adjuvant Targeting ARF6. At the injection site, succinate activates SUCNR1 to trigger a strong immune response; ARF6-ASO specifically silences ARF6 to suppress excessive inflammation. Local administration avoids systemic toxicity, balancing vaccine immunogenicity and safety.

Different from conventional dual-target inhibition schemes, our adjuvant design only targets ARF6 with ASOs. In vaccination sites, succinate is added to activate SUCNR1 for strong immune responses, while ARF6-ASO silences overexpressed ARF6 to eliminate excessive inflammation. This hypothetical design may mitigate the longstanding tradeoff seen with conventional adjuvants, where robust antigen-specific immunity frequently coincides with excessive local inflammatory reactions; direct comparative immunization trials are needed to confirm this predicted balance. Local delivery of ARF6-ASO avoids systemic toxicity, and reserved SUCNR1 activity guarantees vaccine efficacy, which is the core advantage of this novel strategy (Figure 9). Therefore, ARF6/SUCNR1-targeted ASO can balance immune enhancement and safety, which meets the core requirements of novel adjuvants.

### 3.3. Design and Optimization Strategies of ARF6-Targeted ASOs

#### 3.3.1. Sequence Design and Chemical Modification of ASOs

**Figure 10:**
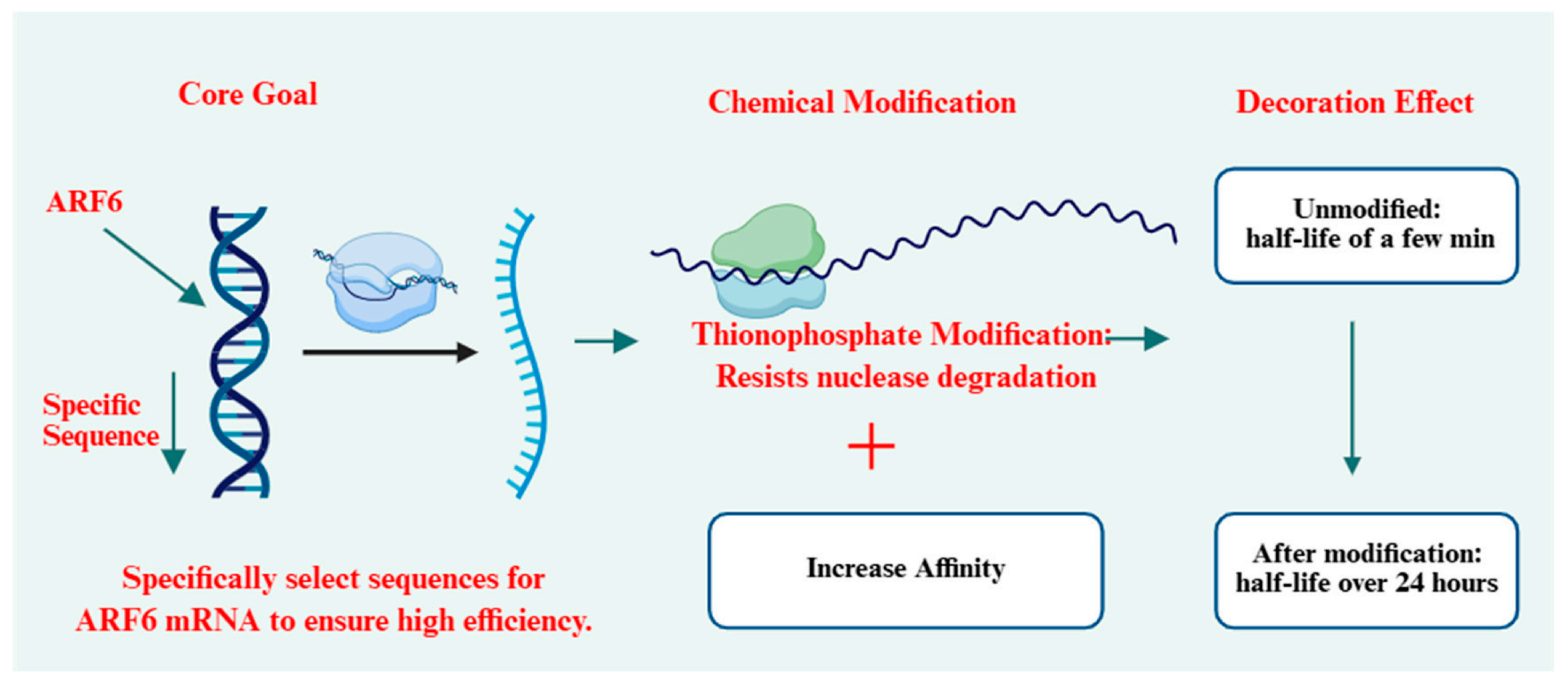
Sequence Design and Chemical Modification Strategies of ARF6 Targeting ASOs. ASO sequence screening and chemical modification. Specific sequences target and silence ARF6 mRNA; dual chemical modifications improve stability and binding ability, greatly extending the in vivo half-life, laying the groundwork for in vivo use.

The core of ASO design is to select specific silencing sequences for ARF6 mRNA to ensure high silencing efficiency. On this basis, chemical modification is required: phosphorothioate modification is used to resist nuclease degradation in vivo; locked nucleic acid (LNA) modification is adopted to improve the binding affinity with mRNA (Figure 10). Reasonable chemical modification can prolong the half-life of ASOs in vivo from several minutes to more than 24 h, laying a foundation for in vivo application.

#### 3.3.2. Tissue/Cell-Targeted Modification for Neutrophil and Immune Tissue Enrichment

**Figure 11:**
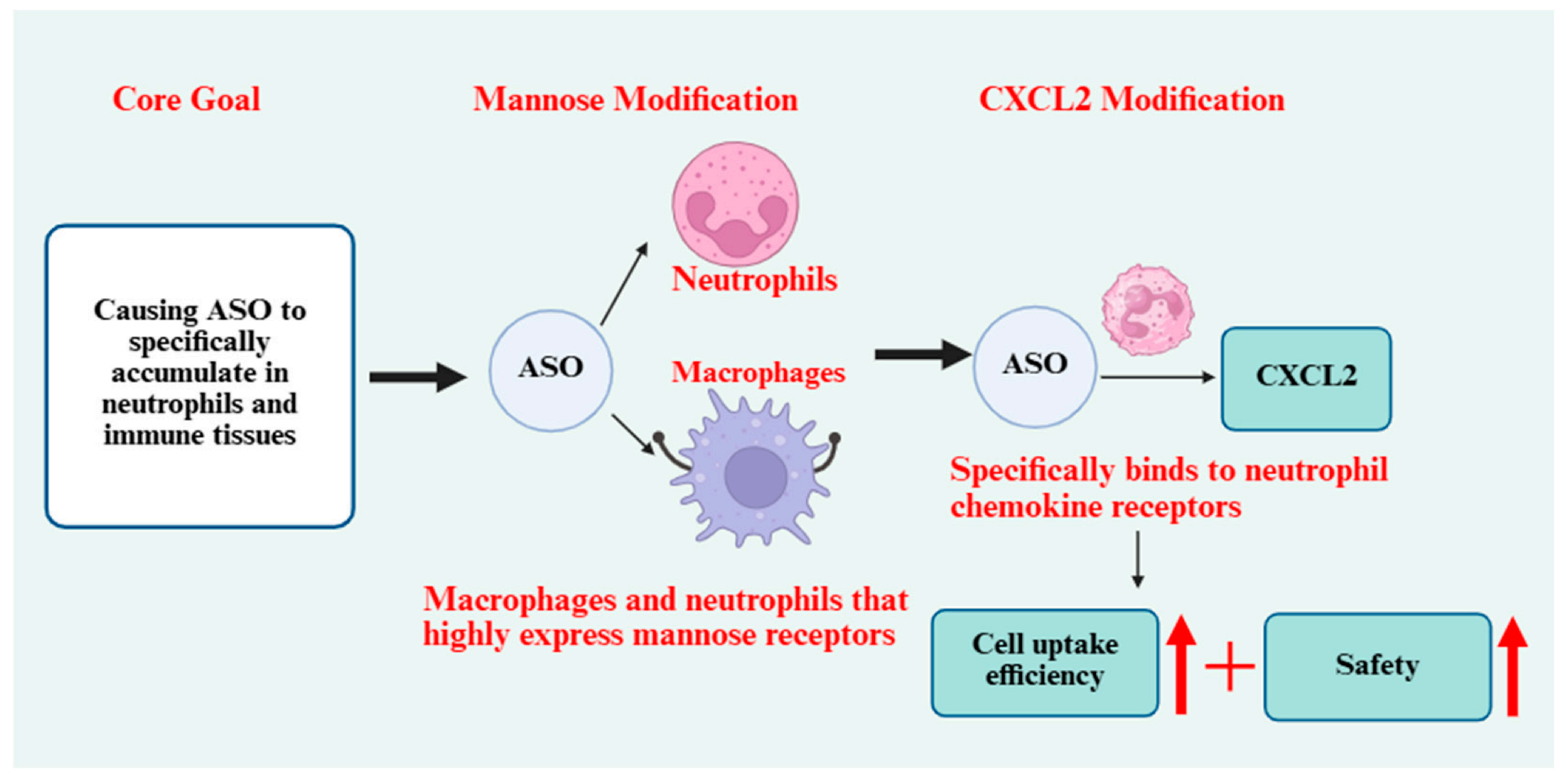
Cell and tissue modification strategies targeting neutrophils for ASO. A cell modification strategy for targeting neutrophils with ASOs. By modifying ASOs with two ligands, mannose and CXCL2, it specifically targets mannose receptor-positive macrophages/neutrophils and neutrophil chemotactic receptors, improving cell uptake efficiency, reducing dosage, and increasing drug safety.

To make ASOs specifically accumulate in neutrophils and immune tissues, targeted ligands can be coupled on the surface of ASOs or nanocarriers: mannose modification can target macrophages and neutrophils highly expressing mannose receptors; CXCL2 fragment modification can specifically bind to neutrophil chemokine receptors, improving cellular uptake efficiency (Figure 11). Targeted modification can further reduce the effective dose of ASO and improve safety.

To restrict ARF6-ASO bioactivity to neutrophil–macrophage populations while reducing off-target silencing in dendritic cells and B lymphocytes, two neutrophil/macrophage-specific surface ligands are conjugated to nanocarrier surfaces: mannose moieties bind mannose receptors highly enriched on myeloid cells, while truncated CXCL2 peptide fragments selectively engage neutrophil CXCR2 chemokine receptors. These dual ligands drive preferential ASO internalization within myeloid effector cells, with negligible carrier uptake by lymphoid B/DC subsets [56].

Even if low-level non-specific ARF6 knockdown occurs in B cells or dendritic cells, limited basal ARF6 suppression only mildly alters constitutive receptor recycling without ablating core antigen presentation or B-cell activation function. The dominant immune priming signal originates from myeloid-derived BAFF secretion downstream of SUCNR1, which remains fully intact independent of minor ARF6 modulation in lymphoid populations. No severe adverse immune suppression is anticipated from limited off-target silencing [9,57].

### 3.4. Combination of ASO with Traditional Vaccine Adjuvants

**Figure 12:**
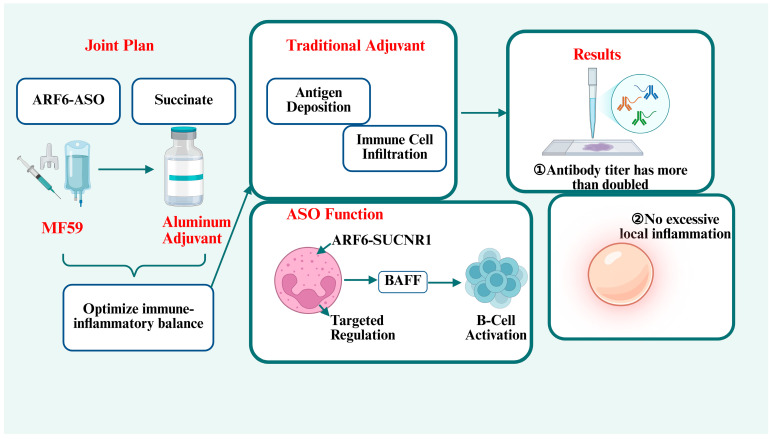
Schematic of ARF6-ASO adjuvant targeting regulation of immune cell activation. ARF6 targets ASO by specifically regulating neutrophils to achieve immune balance, activating immune cells, boosting immune responses, and reducing local inflammation.

Combined with succinate and classic adjuvants, ARF6-ASO further optimizes the immune-inflammatory balance. Traditional emulsified adjuvants (such as MF59) and aluminum adjuvants have good delivery effects and basic immune enhancement ability [55,58], while ASO has targeted regulation on neutrophil function [59]. The combined use of the two can produce synergistic effects: traditional adjuvants promote antigen deposition and local immune cell infiltration, and ASO regulates the ARF6-SUCNR1 axis in terms of recruited neutrophils to amplify BAFF secretion and B-cell activation (Figure 12). Based on separated findings from related preclinical myeloid regulation studies [60,61,62], this triple combination may elevate antigen-specific antibody titers to over two-fold higher levels than single adjuvant formulations while limiting local inflammatory infiltration [63]. Notably, these cited works only validate individual components of our design, and dedicated in vivo immunization trials for the full ARF6-ASO/succinate/classical adjuvant mixture remain unreported to confirm this predictive immunological outcome [9].

### 3.5. Delivery Systems for ARF6-Targeted ASO

All nanodelivery systems in this section are designed for ARF6-targeted ASOs. A major challenge is the ARF6 endocytosis paradox: nanocarrier uptake partially relies on ARF6-mediated pathways [64,65]. We summarize feasible delivery solutions to ensure ARF6-ASO can enter cells normally while exerting inhibitory effects on ARF6.

#### 3.5.1. Key Challenges for In Vivo Delivery of ASO

The in vivo delivery of ASOs faces three major challenges: first, poor stability, which is easily degraded by nucleases in blood and tissue fluid [66,67]; second, low cellular uptake efficiency, and negatively charged nucleic acid has difficulty crossing the cell membrane [67,68]; third, the unique paradox of ARF6 pathway: the cellular uptake of many nanocarriers depends on ARF6-mediated endocytic pathway [69,70]. Systemic silencing of ARF6 will inhibit the entry of nanocarriers, resulting in reduced therapeutic effect [28,69]. Therefore, the design of nanocarriers must consider stability, targeting and avoidance of the ARF6 uptake paradox.

To resolve the first stability limitation, phosphorothioate and LNA modifications introduced in Section 3.3.1 confer intrinsic nuclease resistance to ASOs, while nanocarrier encapsulation further blocks extracellular degradation within injection-site interstitial fluid. For sufficient local tissue penetration, nanocarriers with diameters below 100 nm and PEG-shielded neutral surface charge are theoretically favored, enabling free diffusion through muscle extracellular matrix to reach resident myeloid cells. For controlled intracellular cargo release, all carrier platforms outlined below adopt pH-responsive backbones, which disassemble under acidic endosomal conditions to release ASOs into the cytoplasm and bypass lysosomal degradation [71].

#### 3.5.2. Classic Nanodelivery Platforms for Nucleic Acid Drugs

At present, three types of nanocarriers are widely used for nucleic acid delivery, and each has its own advantages and applicability for ARF6-SUCNR1-targeted ASOs:

- **Lipid nanoparticles (LNPs)**: Mature commercial platform, with good biocompatibility and high nucleic acid loading efficiency [72]. Its native hepatic tropism is a drawback for local vaccination, so surface coupling of neutrophil/macrophage ligands is required to redirect LNPs toward injection-site myeloid cells rather than liver tissue, matching our demand for confined ARF6 knockdown at immunization loci [73,74]. For intramuscular local administration, mannose/CXCL2-modified LNPs may achieve myeloid-specific uptake and support simultaneous co-loading of ASO and succinate. Possibly, LNP uptake relies partially on ARF6-dependent endocytosis, which slightly delays ARF6 knockdown onset; unmodified cationic lipids also trigger mild local carrier-derived inflammation, while hepatic off-target silencing represents its primary translational limitation.
- **Polymeric nanoparticles**: Including PLGA and PEI nanoparticles, with adjustable structure and sustained release function, which can slowly release ASOs in vaccination sites for a long time [75,76]. Cationic polymer cytotoxicity can be mitigated via PEGylation; this optimization also avoids rapid systemic carrier diffusion that would disrupt distant physiological ARF6-SUCNR1 homeostasis [75,76,77].
- **Cell membrane biomimetic vesicles**: Using neutrophil or macrophage membrane to wrap nanocarriers, with immune evasion ability, long circulation time in vivo, and natural targeting to inflammatory foci [78,79,80,81]. This intrinsic myeloid tropism may directly reduce off-target ASO uptake in non-immune cells, fitting our goal to restrict ARF6 silencing to neutrophil–macrophage populations that dominate SUCNR1-mediated adjuvant effects.

#### 3.5.3. Microenvironment-Responsive and Tissue-Targeted Nanocarriers for Neutrophil/Inflammatory Tissue

Inflammatory sites and vaccination sites have unique microenvironmental characteristics such as low pH and high matrix metalloproteinase (MMP) activity. Microenvironment-responsive nanocarriers can remain stable in normal blood circulation and disassemble to release ASO only after entering inflammatory sites, realizing “on-demand release” [82,83,84,85,86,87]. MMP-cleavable linker-modified nanocarriers and pH-sensitive lipid vesicles are the mainstream representatives of such carriers [88,89], which can further improve the local concentration of ASOs and reduce systemic exposure.

#### 3.5.4. Overcoming the “Uptake Paradox” of ARF6 in Nanocarrier Internalization

Aiming at the problem that ARF6 inhibition hinders nanocarrier endocytosis, we propose three coordinated mitigation strategies to resolve this delivery conflict:

Strategy 1: Temporal offset administration of nanocarriers and succinate. Nanocarriers loaded with ARF6-targeted ASOs are administered intramuscularly 6–12 h prior to succinate injection. This time window allows unperturbed ARF6-dependent endocytosis to complete ASO cellular uptake before sufficient ASO accumulates to downregulate pathological ARF6 activity. Basal ARF6 protein existing before ASO silencing mediates full nanocarrier internalization without uptake impairment [90].

Strategy 2: Nanocarrier optimization for ARF6-independent caveolae endocytosis. The nanocarrier formulation is engineered to prioritize caveolae-mediated endocytosis rather than ARF6-dependent macropinocytosis for cellular entry, via surface PEG modification and neutral lipid composition adjustment. Caveolae internalization proceeds independently of ARF6 GTPase activity, decoupling ASO uptake efficiency from subsequent ARF6 protein silencing. This design aligns with our original proposal to select endocytic pathways independent of ARF6 for carrier design, such as caveolae-mediated endocytosis, to avoid relying on ARF6 function [64].

Strategy 3: Local injection to boost passive interstitial diffusion. Local intramuscular injection concentrates nanocarriers within interstitial fluid at vaccination sites, enabling direct cell membrane contact and passive diffusion uptake to further offset any mild reduction in ARF6-mediated vesicular transport. This matches our second core solution: adopt local intramuscular or intraperitoneal injection instead of systemic intravenous injection [91,92]. Local delivery makes ASO and nanocarriers directly act on local cells and avoids the conflict between ARF6 silencing and carrier uptake in distant tissues [90,93]. This is a key technical point for the translational application of ARF6-targeted ASOs.

We acknowledge that the ARF6 endocytosis paradox cannot be completely eliminated under all in vivo conditions, and the above auxiliary temporal design and two core strategies only mitigate rather than fully abolish this conflict. In future preclinical studies, quantitative flow cytometry will be required to quantify ASO cellular uptake efficiency under varying ARF6 knockdown magnitudes, to define the optimal silencing threshold that balances sufficient inflammatory suppression and intact carrier internalization. This unresolved quantitative parameter represents a key knowledge gap summarized in Section 6.1 Future Directions.

## 4. Discussion

ARF6-SUCNR1 crosstalk links metabolism and immunity via neutrophils. Throughout this section, we label all mechanisms as direct paired data, indirect single-gene results, or untested feedback models. SUCNR1 boosts immunity and ARF6 drives inflammation (separate indirect evidence); their bidirectional amplification remains hypothetical [9]. This functional difference makes the traditional single or dual inhibition mode inappropriate for vaccine adjuvant development. Instead, we propose a differentiated regulatory paradigm: retaining and activating SUCNR1 with succinate, while selectively inhibiting ARF6 using ASOs. This hypothetical two-way regulatory framework offers a novel theoretical perspective to bypass persistent drawbacks of single-target adjuvant design, yet all mechanistic and therapeutic claims require dedicated in vitro and in vivo validation.

Antisense oligonucleotides are the optimal tool to realize this one-inhibition-one-activation strategy. Unlike non-selective small-molecule inhibitors, ARF-targeted ASO achieves precise gene silencing without interfering with SUCNR1 expression and function [55]. Combined with various nanocarriers, ARF6-ASO solves the problems of poor stability and low cellular uptake, and the ARF6 endocytosis paradox has also been addressed via rational carrier design and local administration [28,55]. Combined with classic adjuvants and succinate, ARF6-ASO produces synergistic effects, balancing immune enhancement and inflammatory side effects, which outperforms traditional adjuvants in safety and efficacy. For the balancing, it may integrate three core regulatory mechanisms to reconcile the dual physiological and pathological functions of ARF6 and SUCNR1. First, our ASO only triggers partial, transient knockdown of ARF6 restricted to local vaccination sites, instead of complete gene ablation. Residual basal ARF6 activity maintains essential SUCNR1 membrane recycling and baseline neutrophil migratory capacity, while suppressing only excessive ARF6 overactivation induced by succinate-mediated SUCNR1 signaling [94]. Second, local co-delivery of succinate and ARF6-ASO confines SUCNR1 activation within the injection microenvironment and avoids systemic inflammatory spread [9]. Third, partial ARF6 silencing directly breaks the SUCNR1-ARF6 positive feedback loop that amplifies unrestrained tissue inflammation, separating protective B-cell priming signals from harmful neutrophil hyperactivation and NET release. Collectively, these design features enable the strategy to retain adjuvant potency while mitigating the dual inflammatory risks of overactive ARF6 and sustained SUCNR1 stimulation [24,95].

Nevertheless, the prediction that partial ARF6 silencing may preserve neutrophil migratory capacity and sustain intact SUCNR1-dependent B-cell priming remains purely speculative. Independent single-gene knockout data confirm that severe ARF6 depletion blocks actin cytoskeleton rearrangement and abolishes neutrophil directional chemotaxis [5,45]. There exists an uncharacterized narrow therapeutic window between sufficient ARF6 inhibition to curb inflammation and residual ARF6 expression required for normal neutrophil recruitment. No paired succinate + ARF6-ASO animal immunization studies have validated whether this balance can be achieved in actual vaccination microenvironments.

The “inhibit ARF6 + activate SUCNR1” strategy has broad application prospects in prophylactic vaccines and also provides references for the treatment of metabolic and autoimmune diseases [32,96]. This cross-disciplinary scheme combines immunometabolism, nucleic acid therapeutics and nanomedicine. At present, the dose–effect relationship of ARF6-ASO, cell-specific regulation, and long-term biosafety still need further verification. In-depth research on these issues will accelerate the clinical transformation of this novel adjuvant system and promote the innovative development of vaccinology and immunometabolism. Additionally, conventional neutrophil adjuvants including CXCR2 agonists and standalone succinate only modulate one single immune link: they either recruit neutrophils or activate SUCNR1 signaling, but cannot break the ARF6-SUCNR1 inflammatory positive feedback loop. Our proposed two-way differentiated regulation provides a unique theoretical framework to balance adjuvant potency and inflammatory risk, whereas direct experimental comparison will be presented in our subsequent original research upon study completion [9,97].

In summary, the ARF6-SUCNR1 axis plays dual roles in immunity and inflammation. The innovative strategy of using ARF6-targeted ASOs to inhibit excessive ARF6 activity and applying succinate to activate SUCNR1 probably balances vaccine immunogenicity and safety [9,55,98]. Nanocarrier technology guarantees the delivery efficiency of ARF6-ASO and resolves the ARF6 endocytosis paradox [48,55]. This one-inhibition-one-activation paradigm opens a new direction for novel vaccine adjuvant research. Although several challenges remain, further exploration will advance the translational application of this strategy and drive the integrated development of immunometabolism and nucleic acid pharmacy [99,100,101].

## 5. Limitations

Current ARF6 and SUCNR1 research only provides separate single-gene evidence. No direct paired data proves their physical interaction or bidirectional feedback at vaccine injection sites. The proper ARF6 knockdown degree, timing and cell-specific activity threshold remains unmeasured. Most supporting results come from chronic disease models rather than transient vaccination microenvironments. We also lack systematic data on succinate systemic leakage, ASO off-target effects and long-term carrier safety in immunization settings.

## 6. Future Directions

### 6.1. Deciphering the Molecular Mechanism of ARF6-SUCNR1 Crosstalk

Use co-immunoprecipitation, proteomics and other technologies to screen the interactive molecules of the ARF6 and SUCNR1 signaling pathways, clarify the upstream and downstream regulatory network, and analyze the cell-type specific differences of the axis.

### 6.2. Development of Next-Generation Neutrophil-Targeted ASO and Smart Nanocarriers

Optimize ASO chemical modification schemes, develop high-efficiency and low-cost targeted ligands for neutrophils, and design multi-responsive intelligent nanocarriers to further improve targeting and biosafety.

### 6.3. Preclinical and Clinical Evaluation of ASO Adjuvants

Carry out long-term toxicity, immune effect and protection evaluation of ASO adjuvants in multiple animal models [102,103], and gradually promote the transformation from animal experiments to clinical trials on the premise of ensuring safety.

### 6.4. Multi-Omics Strategies to Explore New Regulators of the ARF6-SUCNR1 Axis

Combined with transcriptomics, metabolomics and other multi-omics technologies, discover new regulatory molecules of the ARF6-SUCNR1 axis, expand the target library of adjuvant development.

### 6.5. Autoimmune Inflammatory Diseases

In autoimmune diseases, inhibiting SUCNR1 with specific inhibitors can reduce neutrophil infiltration, which is another independent application direction, not the focus of this review.

## 7. Conclusions

Existing studies separately confirm ARF6 drives local inflammation and SUCNR1 boosts neutrophil-dependent humoral immunity. The ASO–succinate dual-regulation design is only a testable theoretical idea, not a validated adjuvant solution. Nanocarriers enable local co-delivery of two agents but each platform carries unique targeting, silencing speed and inflammatory drawbacks. All ARF6-SUCNR1 reciprocal crosstalk and the balance effect of partial ARF6 silencing remain unvalidated. Major unsolved mechanistic and safety questions are summarized in Section 5, and comprehensive preclinical trials are necessary to judge the practical value of this framework.

## Data Availability

No new data were created or analyzed in this study. Data sharing is not applicable to this article.
